# Commute patterns and depression: Evidence from eleven Latin American cities

**DOI:** 10.1016/j.jth.2019.100607

**Published:** 2019-09

**Authors:** Xize Wang, Daniel A. Rodríguez, Olga L. Sarmiento, Oscar Guaje

**Affiliations:** a Department of Real Estate, National University of Singapore, Singapore; b Department of City and Regional Planning, University of California, Berkeley, USA; c School of Medicine, Universidad de Los Andes, Bogotá, Colombia; d Department of Industrial Engineering, Universidad de Los Andes, Bogotá, Colombia

**Keywords:** Transportation, Commute, Mental health, Depression, Latin America, Random effects model

## Abstract

**Introduction:**

Although travel behavior is expected to influence personal health, few studies have examined associations with mental health. This study examines associations between commute patterns and mental health using survey data in 11 Latin American cities.

**Methods:**

Using a survey conducted by the Development Bank of Latin America in 2016, we measured the presence of depressive symptoms using the 10-item Center for Epidemiologic Studies Depression (CESD-10) screening scale. We used multilevel non-linear models to estimate the magnitude of the associations between commute patterns and depression risk, adjusting for socio-demographic and neighborhood characteristics.

**Results:**

We found that, on average, every 10 more minutes of commuting time is associated with 0.5% (p = 0.011) higher probability of screening positively for depression. Furthermore, when decomposing commuting time into free-flow time and delay time, we found that delay and not free-flow time, were associated with depression. Specifically, every 10 additional minutes of traffic delay is associated with 0.8% (p = 0.037) higher probability of screening positively for depression. When examining differences by travel mode, we find that users of formal transit (e.g. subway or bus rapid transit) are 4.8% (p = 0.040) less likely to be screened positively for depression than drivers. In addition, not having transit stops within a 10-min walk from home is associated with higher probability of screening positively for depression.

**Conclusions:**

Our findings provide preliminary evidence that better access to mass transit systems and less congestion may be linked to better mental health among urban residents.

## Highlights

• Longer commuting time is associated with higher probability of depression.
• Traffic delay, not uncongested travel time, is associated with depression.
• Commuters using formal transit have lower probability of depression than drivers.
• Poor access to transit is associated with higher probability of depression.
• Congestion relief and transit coverage expansions can have mental health benefits.

## Resumen

**Introducción:**

Aunque se espera que el comportamiento de viajeros afecte su salud personal, pocas investigaciones han estudiado el efecto de estos comportamientos sobre la salud mental. En este estudio examinamos asociaciones entre patrones de viaje y salud mental en individuos de 11 ciudades de América Latina.

**Métodos:**

Utilizamos una encuesta del Banco de Desarrollo de América Latina (CAF) del 2016, para medir la presencia de síntomas depresivos con la escala de tamizaje de depresión del Center for Epidemiologic Studies (CESD-10). Usamos modelos de multinivel no-lineales para estimar asociaciones entre patrones de viaje y riesgo de depresión, ajustando por factores socio-demográficos individuales y características del vecindario de residencia.

**Resultados:**

Encontramos que en promedio, cada 10 minutos adicionales de tiempo de viaje están asociados con un aumento de 0.5% (p = 0.011) en la probabilidad de presentar síntomas depresivos de acuerdo con la prueba de tamizaje. Al descomponer el tiempo de viaje en tiempo de demora por congestión y tiempo de viaje sin congestión, encontramos que el tiempo de demora por congestión está asociado con síntomas depresivos, más no el tiempo sin congestión. Específicamente, cada 10 minutos adicionales de tiempo de demora están asociados con 0.8% (p = 0.037) mayor probabilidad de presentar síntomas depresivos. Cuando comparamos los resultados por medio de transporte utilizado, encontramos que usuarios del sistema de transporte público formal (p.ej. metro o buses rápidos) tienen una probabilidad 4.8% (p = 0.040) menor de presentar estos síntomas depresivos con relación a conductores de vehículos privados. Además, no tener paradas de transporte público (formal o informal) a diez minutos o menos a pie del lugar de residencia está asociado con una probabilidad más alta de presentar síntomas depresivos.

**Conclusiones:**

Esta es evidencia preliminar que muestra que la utilización de transporte masivo, mejor acceso a éste, y menor congestión están asociados a mejor salud mental de los residentes de la ciudad.

## Resumo

**Introdução:**

Padrões de deslocamento influenciam a saúde pessoal do indivíduo, porém, poucos estudos examinaram suas associações com a saúde mental. Este estudo examina associações entre padrões de deslocamento e saúde mental usando dados de inquéritos realizados em 11 cidades da América Latina.

**Métodos:**

Utilizando uma pesquisa realizada pelo Banco de Desenvolvimento da América Latina em 2016, medimos a presença de sintomas depressivos usando a escala de triagem do Centro de Estudos Epidemiológicos de Depressão (CESD-10), com 10 itens. Utilizamos modelos multiníveis não lineares para estimar a magnitude das associações entre os padrões de deslocamento e o risco de depressão, ajustando para características sociodemográficas e de vizinhança.

**Resultados:**

Verificou-se que, em média, cada 10 minutos a mais no tempo de deslocamento está associado a 0,5% (p = 0,011) maior probabilidade de rastreamento positivo para depressão. Além disso, ao decompor o tempo de deslocamento entre tempo de fluxo livre e tempo de atraso, descobrimos que o atraso e não o tempo de fluxo livre estava associado à depressão. Especificamente, cada 10 minutos adicionais de atraso no trânsito está associada a 0,8% (p = 0,037) maior probabilidade de rastreamento positivo para depressão. Ao examinar as diferenças por modo de viagem, descobrimos que os usuários de transporte público (por exemplo, metrô ou Transporte Rápido por Ônibus-BRT) têm 4,8% (p = 0,040) menos probabilidade de serem testados positivamente para depressão do que os motoristas. Além disso, não haver paradas de transporte público em um raio de caminhada de 10 minutos a partir do local de moradia também é associado com maior probabilidade de rastreio positivo para depressão.

**Conclusões:**

Nossos resultados fornecem evidências preliminares de que um melhor acesso a sistemas de transporte público e menos congestionamento podem ser fatores associados a uma melhor saúde mental entre os residentes de áreas urbanas.

## 1 Introduction

Mental health is an important component of individual functioning and subjective well-being (Dickerson et al., 2014), and has been included in the UN Sustainable Development Goals (United Nations, 2015). Despite its importance, mental health is emerging as a recurrent societal concern. Mental disorders including depression and anxiety make a significant contribution to the burden of disease and disability in low- and middle-income countries (LMIC) (Lopez et al., 2006; World Health Organization (WHO), 2011). In Latin America, over 20% of the disease burden, measured by disability-adjusted life years, can be attributed to neurological or mental disorders (Sampson et al., 2019). Such a high prevalence of mental health issues raises important concerns regarding well-being and livability, in addition to creating a heavy financial burden to individuals and society.

Mental health is significantly shaped by environmental factors. Specifically, transportation as a social determinant of health is likely to affect mental health (Marmot, 2005). For drivers, stress from traffic, travel delay uncertainty, and long travel times are likely to impact mental health (e.g. (Martin et al., 2014)). For transit users, concerns about service reliability and personal safety, and limited comfort may affect mental health (e.g. (Evans et al., 2002)). Despite these expected associations, there is limited evidence examining associations between mental health and commuting, especially in Latin American cities. This study focuses on a particular aspect of mental health: depression, and examines associations between depression risk and commuting patterns – travel time, traffic delay, and travel modes – in a sample of residents of 11 cities in Latin America: Buenos Aires, Bogotá, Caracas, Fortaleza, La Paz, Lima, México City, Montevideo, Panamá City, Quito and Sao Paulo.

This study contributes to the literature in three ways. First, we contribute towards disentangling the relationship between commute duration and depression by separately examining the effects of travel delay time induced by congestion from uncongested travel time. Second, we control for a comprehensive list of covariates, especially neighborhood characteristics, which have not been accounted for in most previous studies. And third, we extend the geographic coverage of prior research to include Latin America, and covering multiple cities with significant variation in geographic and socio-economic characteristics.

## 2 Commuting and mental health

The theoretical foundations connecting commuting behavior with mental health originate in “impedance theory” by Novaco et al. (1979). Novaco et al. (1979) defined “impedance” as the difficulty in moving from the origin to the destination of a commuting trip. Specifically, physical impedance measures the distance and time needed to complete a trip, while subjective impedance indicates the personal effort required by a person to travel (Novaco et al., 1990). Both physical and subjective impedance create stress through the frustration of failing to achieve the task (Novaco et al., 1979). This link between impedance and stress has been demonstrated by a large literature (Avila-Palencia et al., 2017; Evans and Wener, 2006; Gatersleben and Uzzell, 2007; Legrain et al., 2015; Ruger et al., 2017; Wener et al., 2003; Wener and Evans, 2011). Moreover, people constantly exposed to stressful events are more likely to have depressive symptoms and worse mental health conditions (Turner et al., 1995).

Longer commuting duration has been associated with poorer mental health. For instance, using a national-level, longitudinal dataset in UK (British Household Panel Survey), Feng and Boyle (2014) found out that men commuting for a longer time had worse mental health. Using the same dataset, Martin et al. (2014) found that those spending more time driving often suffer from more severe mental health issues. However, the association becomes less clear when adjusting for socio-demographic characteristics (Dickerson et al., 2014; Hansson et al., 2011).

Congestion has also been associated with adverse mental health outcomes due to its potential impact on sense of control (Schaeffer et al., 1988), lower predictability of the trip duration (Kluger, 1998) and a higher level of fatigue (Costa et al., 1988). In addition, the relationship between commuting and mental health may be mediated by time urgency (Hennessy and Wiesenthal, 1999; Koslowsky, 1997) and personality traits (Stokols et al., 1978).

Commuters using mass transit and active travel modes tend to have better mental health conditions than those driving to work. For instance, Mytton et al. (2016) found that those commuting by transit, bicycling and walking report lower levels of mental distress than drivers. Similar patterns are also found in school children in China (Sun et al., 2015). Knott, Panter, Foley, and Ogilvie (2018) found that drivers in UK report less severe depressive symptoms after shifting to active commuting modes of travel. Active commuters also have higher level of mindfulness than automobile commuters (LaJeunesse and Rodríguez, 2012). However, others have found that spending longer time walking or bicycling is not associated with mental health (Humphreys et al., 2013; Kuwahara et al., 2015). In addition, factors such as transit service quality, and transit connectivity associated with better mental health (Yang et al., 2018; Chng et al., 2016).

In summary, although theoretical and empirical evidence regarding associations between commuting patterns and mental health is emerging, significant questions remain. It is abundantly clear that socio-demographic characteristics confound associations between travel patterns and mental health. Yet, studies that have accounted for the socio-demographic characteristics of participants have failed to account other factors – such as neighborhood characteristics – that are likely to explain both commuting behaviors and mental health outcomes (e.g. Kim, 2008). Furthermore, the overwhelming focus on commute duration obscures our ability to understand the degree to which travel delay from congestion, versus sheer commuting time due to long travel distances, are responsible for associations observed. This distinction is important as both commuting distances and congestion continue to increase in many cities worldwide, even though policies aimed at addressing the root cause may differ. Thus, attempting to disentangle the effects of travel delay due to congestion from the effects of uncongested commute times will help clarify causal pathways in the associations identified previously. Finally, except for a few studies using a nationally-representative dataset from the UK (e.g. Martin et al. (2014) and Knott et al. (2018)), most studies focus on a small geographic area in North America, Western Europe or East Asia. Studies in Latin American cities are still very rare. This study aims to fill these gaps by examining associations between commuting patterns and mental health among survey respondents in 11 Latin American cities.

## 3 Data and methods

### 3.1 Survey data

Data for this study come from a survey conducted by the Development Bank of Latin America (*Corporación Andina de Fomento* or CAF) in 2016. The survey (*La Encuesta* CAF 2016 or ECAF 2016) includes cross-sectional data from 12,905 individuals in 11 Latin American cities collected between November 2016 and January 2017 (Development Bank of Latin America, 2017). Cities included are: Buenos Aires (Argentina), Bogotá (Colombia), Caracas (Venezuela), Fortaleza (Brazil), La Paz (Bolivia), Lima (Perú), México City (México), Montevideo (Uruguay), Panamá City (Panamá), Quito (Ecuador) and Sao Paulo (Brazil). Sampling was stratified by city and was intended to produce representative results for the population in each city. The sampling details are summarized elsewhere (Development Bank of Latin America, 2017). For each household, only one individual (20–60 years old) was interviewed.

In four out of the 11 cities (Buenos Aires, Bogotá, Caracas and Fortaleza), the surveys were further stratified by whether respondents reside in formal or informal settlements. Informal settlements were defined as neighborhoods with more than 50 contiguous houses with no title, with no formal access to utilities, or with significant building deficiencies (Development Bank of Latin America, 2017). For each city, the survey included about 1,000 individuals residing in formal neighborhoods except for Panamá City which only had 600 respondents; for the four cities where informal neighborhood dwellers were sampled, an additional 500 individuals living in those neighborhoods were also included.

### 3.2 Outcome variable

Depression was measured using the ten-item Center for Epidemiologic Studies (CESD-10) scale (Andersen et al., 1994). The CESD-10 scale has been shown to be a valid and reliable screening tool for depression both in Latin America and in other regions (Andersen et al., 1994; Bradley et al., 2010; Schantz et al., 2017). The scale includes a list of ten items on feelings (for instance, *“I was disturbed by things that do not usually bother me”*). Each individual is asked about how often in the past week he/she has each of these feelings. The responses are then converted to a 0–30 score, with higher values indicating a higher likelihood of depressive symptoms. A person with a CESD-10 score of 10 or higher is categorized as positive for the depression screen. Hence, we use the scale to create a dummy variable which equals one if the individual screens positively for depression and zero otherwise (“depression” hereafter for simplicity). All respondents with up to two items missing in the scale were included. For those respondents, the scores of their missing items were imputed using the mean score of the available items. We are more tolerant compared to Andersen et al. (1994), who only allowed up to one item missing. We conducted a sensitivity analysis to assess both approaches (one missing vs. two missing scale items) as well as the list-wise deletion approach (i.e. only no missing scale items); the results from these analyses did not differ.

### 3.3 Overall commuting time, traffic delay and modes

Travel-related variables include door-to-door commuting time during a “normal day,” uncongested commuting time, and travel mode(s) commonly used for the commute, all self-reported. Specifically, the commute time variables are defined as “on average, the travel time from home to the place where one carries out the main daily activity”, and therefore this definition does not specify whether the “main activity” is work, school or others. To reduce the potential influence of unrealistic commutes, we truncated those who reported travel time of more than 180 min into 180 min (N = 36). We truncated both overall commute time and uncongested travel time. We also estimated travel delay time during commuting by subtracting commuting time from commuting time without traffic. Furthermore, we excluded 23 respondents whose delay-to-overall-time ratio was larger than 90%. Sensitivity analyses to the thresholds for truncation of travel time and exclusion for excessive delay-to-overall travel time were also conducted. To determine commute mode(s) used, each respondent selected one or more among 14 alternatives. From this we created a categorical variable with six mutually-exclusive groups identifying the primary mode used: private automobile, formal transit, informal transit, taxi/shuttle, non-motorized, and other.^1^ We also conducted a sensitivity analysis to our definition of main commuting travel modes.

### 3.4 Control variables

Control variables include 10 individual socio-demographic variables and 11 variables measuring neighborhood physical and social characteristics. Socio-demographic variables include gender (male/female), age, living with spouse (yes/no), having children (yes/no), employed (yes/no), education (less than high school, completed high school or some college; completed college or higher), home ownership (yes/no), whether the respondent had moved in the past five years (yes/no), automobile ownership (yes/no), and motorcycle ownership (yes/no). Unfortunately, ECAF 2016 does contain information on total household income. Self-reported variables measuring neighborhood characteristics include presence of frequent violent crimes (yes/no) and having poorly lit streets (yes/no) within the neighborhood, presence of abandoned buildings (yes/no) and landfills (yes/no) within three blocks of the respondent’s residence, having sidewalks within a block (yes/no), and having at least a school (yes/no), hospital (yes/no), park (yes/no), and lack of transit (yes/no) within a 10-min walk from the residence. Variables observed by surveyors include whether the residence is a detached house (yes/no), and whether the neighborhood qualifies as informal (yes/no) using the aforementioned definition.

### 3.5 Validation of self-reported overall and uncongested commuting time

Since our exposures are self-reported, it is important to evaluate the degree of validity of such data. To this end, for a subsample of 269 participants of the study sample in Bogotá, we geocoded their home origin and their main work/school destination. To preserve confidentiality, geocodes were aggregated to 500m by 500m gridcells. We then used the Google Maps API (Google, n.d.) to measure morning peak hour (7:30am) and uncongested travel time between each origin-destination pair for each respondent. Measurements were made for 15 weekdays over three weeks (Tuesday through Thursday) in the morning peak and at midnight by automobile during May, 2018. The city of Bogotá has an average temperature of 14 °C with alternate periods of rain and drought rather than seasons. The average temperature in May is comparable with that in January (when the survey data is collected), while the average precipitation in May is higher than that in January. We compare the median congested and uncongested travel time to self-reports for each individual. In every case we only estimate automobile travel times, and thus assume that automobile travel time is an appropriate surrogate for transit travel time. Since Bogotá’s bus rapid transit system has a vast network of bus rapid transit (BRT)-only lanes, our analysis is likely to yield lower agreement for transit users. For the validation of self-reported congested and uncongested travel time, we use Bland-Altman plots (Bland and Altman, 1986; Giavarina, 2015) as well as Pearson’s correlation index.

### 3.6 Statistical modeling

Since the dependent variable is binary, we used multilevel logistic regression and included a random effect at the city level to account for heterogeneity across cities. We estimated two sets of models. In the first we estimated associations between overall commuting time and depression. In the second set of models, we focused on the two components of overall commuting time: traffic delay time and uncongested commute time. We estimated a total of eight models, four in each set. For each set, we first estimated unadjusted associations while allowing for city-specific random effects. Then we incrementally added three blocks of variables (main travel mode, individual socio-demographic characteristics, and neighborhood characteristics) so that the fourth model in each set includes fully adjusted associations. We estimated variance inflation factors (VIF) for the fully-adjusted models and did not find concerns with multicollinearity. We also compared the mean values of the outcome, exposure and control variables for those in the estimation sample and those excluded (when available). All analyses were conducted in Stata 15 (StataCorp., 2017).

## 4 Results

### 4.1 Descriptive statistics

The final sample in the study includes 5,438 individuals. These individuals have complete information on depression risk, commuting patterns, and socio-demographic and neighborhood characteristics. A total of 7,467 individuals (out of the total 12,905 in the original ECAF 2016 survey) were excluded from the analysis because 4,555 do not have complete information on depressive symptoms, 1,093 do not report full commuting patterns, 460 do not have complete data on all individual socio-demographic characteristics, and 1,323 do not have complete neighborhood characteristics, and 36 have a delay-to-overall-time ratio larger than 90%. Table 1 shows that commute patterns, socio-demographic characteristics and neighborhood characteristics of those included in the estimation sample and those excluded are comparable. Table 1 also shows that the share of individuals having depression in the estimation sample is smaller than those excluded. However, note that for individuals who were excluded, only 18% of them have complete CES-D information, a fairly small number.

**Table 1:** Comparing individuals in and out of the study sample.

Variable	In sample		Out of sample		Difference (in sample – out of sample)		
	mean	N	mean	N	diff	%	p-value
***Dependent variable***							
Screening positively for depression (1/0)	0.37	5438	0.43	1926	−0.05	−12.8%	<0.001
***Commuting patterns***							
Overall commuting time (10 min units)	3.88	5438	3.75	5777	0.13	3.4%	0.051
Traffic delay time (10 min units)	1.34	5438	1.31	5777	0.03	2.3%	0.364
Uncongested travel time (10 min units)	2.54	5438	2.44	5777	0.10	3.9%	0.026
Commute mode (%)							
*Private automobile*	11.0	5438	11.27	5583	−0.27	−2.4%	0.653
*Formal transit*	47.79	5438	49.81	5583	−2.02	−4.1%	0.034
*Informal transit*	8.33	5438	9.22	5583	−0.89	−9.7%	0.097
*Taxi/Uber/shuttle*	3.31	5438	3.92	5583	−0.61	−15.6%	0.085
*Non-motorized*	0.24	5438	0.20	5583	0.04	21.4%	<0.001
*Other*	5.87	5438	6.25	5583	−0.38	−6.2%	0.397
***Socio-demographic characteristics***							
Female	0.52	5438	0.51	5777	0.01	1.2%	0.527
Age (years)	36.72	5438	37.35	5776	−0.63	−1.7%	0.005
Living with spouse	0.59	5438	0.61	5689	−0.02	−3.1%	0.046
Having children	0.72	5438	0.73	5766	−0.01	−2.0%	0.077
Employed	0.67	5438	0.65	5689	0.01	2.2%	0.103
Education (%)							
*Less than high school*	39.70	5438	42.17	5770	−2.46	−5.8%	0.008
*High school/some college*	49.91	5438	48.34	5770	1.57	3.3%	0.096
*College or higher*	10.39	5438	9.50	5770	0.89	9.4%	0.114
Homeowner	0.66	5438	0.67	5702	−0.02	−2.3%	0.077
Moved in the past five years	0.33	5438	0.33	5651	0.00	0.4%	0.896
Own automobiles	0.32	5438	0.32	5476	0.00	0.9%	0.756
Own motorcycles	0.01	5438	0.01	5414	0.00	−14.3%	0.347
***Neighborhood characteristics***							
Frequent violent crimes in neighborhood	0.50	5438	0.47	5706	0.03	6.0%	0.003
Abandoned buildings within three blocks	0.31	5438	0.28	5473	0.03	10.7%	0.001
Landfills within three blocks	0.33	5438	0.31	5689	0.01	3.7%	0.191
Poorly lit street within three blocks	0.51	5438	0.47	5699	0.04	8.3%	<0.001
Hospitals within 10 min’ walk	0.36	5438	0.39	5683	−0.02	−6.2%	0.009
Schools within 10 min’ walk	0.58	5438	0.60	5690	−0.03	−4.6%	0.003
Sidewalks within block	0.71	5438	0.70	5777	0.01	1.6%	0.187
Living in detached house	0.83	5438	0.83	5762	0.00	0.0%	0.988
Parks within 10 min’ walk	0.54	5438	0.57	5490	−0.03	−5.0%	0.003
Living in informal settlements	0.20	5438	0.15	5777	0.04	28.7%	<0.001
No formal/informal transit stops within 10 min’ walk	0.15	5438	0.12	3591	0.03	28.6%	<0.001

Note: the sample of study only includes individuals with a delay-to-overall-time ratio 90% or lower; similarly, any individuals with delay-to-overall-time ratio larger than 90% were excluded in the out-of-sample comparison group.

Descriptive statistics show that 37% of the study sample screened positively for depressive symptoms (Table 2). In terms of travel, average commuting time is 39 minutes and average traffic delay is 13 minutes, but both exhibit considerable variation in the sample. When examined by city, the cities with the highest overall commuting time are Bogotá and México City, while the cities with the highest traffic delay time are Panamá City and Bogotá. The most popular mode of travel to work or school is formal transit, with a share of 47.8%, followed by non-motorized (23.7%), and private automobile (11.0%). For mode share, Quito showed the highest modal share for formal transit (75.6%) and La Paz showed the lowest (6.2%). For private automobile, Panamá City had the highest share (17.6%) and Lima had the lowest (3.9%). Overall, 52% of respondents are female, 59% live with spouse, 60% have at least a high school diploma, and slightly under one third owns at least one automobile. As to neighborhood patterns, half of the sample live in neighborhoods with frequent violent crimes, 20% live in informal settlements, and 15% do not have access to transit within 10 min of their home.

**Table 2:** Characteristics of the study sample, stratified by cities (n = 5438).

Variable	Mean or %											
	All	BA	LAP	SP	FOR	BOG	QUI	LIM	MVD	CCS	PAC	MEX
***Dependent variable***												
Screening positively for depression (1/0)	0.37	0.27	0.51	0.50	0.52	0.36	0.42	0.40	0.22	0.43	0.34	0.29
***Commuting patterns***												
Overall commuting time (10 min units)	3.88	3.34	3.48	3.56	3.37	5.68	4.48	3.11	2.68	3.82	4.49	5.05
Traffic delay time (10 min units)	1.34	0.82	1.29	1.19	1.08	2.19	1.78	1.15	0.77	1.14	2.22	1.80
Uncongested travel time (10 min units)	2.54	2.51	2.19	2.37	2.29	3.49	2.70	1.95	1.91	2.68	2.27	3.24
Commute mode (%)												
*Private automobile*	11	9.3	7.3	16.7	10.8	11.6	8.2	3.9	13.3	10.1	17.6	16.7
*Formal transit*	47.8	49.5	6.2	44.2	47.5	52.8	75.6	39.8	47.7	61.8	56.6	57.1
*Informal transit*	8.3	0.3	63.1	0.8	3.6	1.2	2.2	12.3	3.0	4.2	4.4	4.1
*Taxi/Uber/shuttle*	3.3	1.3	3.5	3.4	2.2	3.7	3.5	5.3	1.5	0.6	13.2	5.7
*Non-motorized*	23.7	36.1	18.5	30.3	23.0	24.0	8.9	22.6	28.3	17.7	7.7	14.2
*Other*	5.9	3.6	1.3	4.6	13.0	6.8	1.6	16.1	6.2	5.7	0.6	2.2
***Socio-demographic characteristics***												
Female	0.52	0.50	0.50	0.50	0.49	0.61	0.51	0.51	0.55	0.53	0.40	0.47
Age (years)	36.7	37.0	34.1	36.4	36.7	38.3	35.2	36.2	37.6	36.9	37.3	36.3
Living with spouse	0.59	0.63	0.55	0.56	0.59	0.59	0.61	0.59	0.56	0.59	0.55	0.61
Having children	0.72	0.78	0.64	0.70	0.76	0.75	0.74	0.72	0.69	0.75	0.70	0.62
Employed	0.67	0.69	0.69	0.68	0.84	0.66	0.58	0.62	0.66	0.68	0.74	0.62
Education (%)												
*Less than high school*	39.7	60.9	17.9	35.1	68.4	39.8	54.1	19.0	55.1	25.7	26.9	41.0
*High school/some college*	49.9	37.3	62.7	53.9	28.8	46.1	39.6	71.2	35.5	64.3	48.4	49.2
*College or higher*	10.4	1.9	19.4	11.0	2.9	14.1	6.3	9.8	9.3	10.1	24.7	9.8
Homeowner	0.66	0.69	0.55	0.61	0.63	0.63	0.53	0.70	0.59	0.84	0.71	0.78
Moved in the past five years	0.33	0.23	0.37	0.38	0.30	0.43	0.42	0.23	0.48	0.12	0.45	0.15
Own automobiles	0.32	0.30	0.32	0.51	0.17	0.27	0.28	0.18	0.37	0.23	0.43	0.48
Own motorcycles	0.01	0.01	0.02	0.01	0.01	0.01	0.02	0.00	0.01	0.01	0.03	0.03
***Neighborhood characteristics***												
Frequent violent crimes in neighborhood	0.50	0.63	0.44	0.50	0.63	0.43	0.41	0.51	0.55	0.55	0.30	0.42
Abandoned buildings within three blocks	0.31	0.29	0.32	0.28	0.18	0.37	0.54	0.28	0.43	0.07	0.33	0.25
Landfills within three blocks	0.33	0.37	0.49	0.14	0.19	0.36	0.33	0.22	0.38	0.52	0.23	0.16
Poorly lit street within three blocks	0.51	0.69	0.64	0.33	0.37	0.50	0.51	0.37	0.48	0.61	0.37	0.58
Hospitals within 10 min’ walk	0.36	0.58	0.34	0.25	0.53	0.22	0.29	0.41	0.45	0.30	0.30	0.34
Schools within 10 min’ walk	0.58	0.79	0.43	0.54	0.77	0.47	0.36	0.64	0.74	0.53	0.42	0.50
Sidewalks within block	0.71	0.73	0.81	0.98	0.88	0.52	0.89	0.85	0.83	0.47	0.59	0.23
Living in detached house	0.83	0.88	0.88	0.85	0.96	0.77	0.84	0.86	0.81	0.73	0.77	0.83
Parks within 10 min’ walk	0.54	0.65	0.50	0.37	0.58	0.52	0.48	0.67	0.77	0.35	0.51	0.46
Living in informal settlements	0.20	0.58	0.00	0.00	0.40	0.48	0.00	0.00	0.00	0.34	0.00	0.00
No formal/informal transit stops within 10 min’ walk	0.15	0.08	0.25	0.13	0.05	0.25	0.22	0.12	0.05	0.23	0.12	0.11

Number of observations	5438	754	453	647	139	830	316	610	664	526	182	317

Note: Acronyms for cities: BA – Buenos Aires, LAP – La Paz, SP – Sao Paulo, FOR – Fortaleza, BOG – Bogotá, QUI – Quito, LIM – Lima, MVD – Montevideo, CCS – Caracas, PAC – Panamá City, MEX – México City. Mode options to select from included: (1) private automobile; (2) firm or educational establishment transportation; (3) minibus/jeeps/combis; (4) taxi; (5) Uber/app-based ride hailing; (6) motorcycle or bicycle taxi; (7) walking; (8) bicycle; (9) motorcycle; (10) subway; (11) suburban train; (12) buses/coaches (formal); (13) BRT; (14) others. To create the categorical variable, we have used the following rule: (a) if 10, 11, 12 or 13: “formal transit”, otherwise, (b) if 3: “informal transit”, otherwise, (c) if 2, 4, or 5: “taxi/Uber/shuttle”, otherwise, (d) if 1: “private automobile”, otherwise, (e) if 7 or 8: “non-motorized”, otherwise: “other”.

### 4.2 Commuting patterns and depressive symptoms

The first set of models suggest that individuals with longer overall commuting time are more likely to be screened positively for depression (Table 3). Based on the fully-adjusted model (Model 4), holding continuous covariates at their mean values and non-continuous covariates at their modes, every additional 10 min of commuting is associated with a 0.5% higher probability of depression. When separating out the effects of delayed vs uncongested travel time components, we find that it is traffic delay time, as opposed to uncongested travel time, that is significantly associated with depression (Table 4). Based on the fully adjusted model (Model 8), an additional 10 min of traffic delay time is associated with 0.8% higher probability of having depression. Although the coefficients of overall commuting time vary across Models 1–4, their 95% confidence intervals overlap with each other (Table 3). Similarly, the 95% confidence intervals for the coefficients of traffic delay time from Models 5–8 also overlap (Table 4). This suggests that the associations of commuting time and traffic delay do not differ with or without control variables. For both fully adjusted models (Model 4 and Model 8), 5.5% (95% CI: 2.3%–12.7%) of the total variation was due to heterogeneity across cities (Table 3, Table 4). Models with additional interaction terms show that the associations between overall commute time/traffic delay time and depression are not moderated by gender or commute modes. We interacted uncongested travel time and traffic delay and did not find a statistically significant relationship between this interaction term and depression.

**Table 3:** Associations between commuting time and self-reported depressive symptoms (n = 5438).

Depressive symptoms	(1)	(2)	(3)	(4)
	Unadjusted	(1) + modes	(2) + socio-demographics	(3) + neighborhood characteristics
***Commuting patterns***				
Commuting time (in 10 min)	0.015*	0.024**	0.028***	0.026**
	[-0.002,0.031]	[0.006,0.043]	[0.009,0.047]	[0.006,0.046]
Commuting modes				
*Private automobile*		(ref.)	(ref.)	(ref.)

*Formal transit*		0.065	−0.186	−0.242**
		[-0.132,0.262]	[-0.411,0.039]	[-0.470,-0.013]
*Informal transit*		0.241	0.003	−0.067
		[-0.053,0.535]	[-0.314,0.319]	[-0.389,0.255]
*Taxi/Uber/Shuttle*		0.247	0.096	−0.000
		[-0.103,0.598]	[-0.267,0.459]	[-0.371,0.371]
*Non-motorized*		0.255**	−0.051	−0.110
		[0.043,0.468]	[-0.293,0.191]	[-0.357,0.136]
*Other*		0.227	0.035	−0.081
		[-0.066,0.520]	[-0.279,0.349]	[-0.402,0.240]
***Socio-demographic characteristics***				
Female			0.366***	0.358***
			[0.241,0.490]	[0.231,0.484]
Age			0.045**	0.032*
			[0.007,0.082]	[-0.006,0.071]
Age squared			−0.0005**	−0.0003
			[-0.001,0.000]	[-0.001,0.000]
Living with spouse			−0.330***	−0.331***
			[-0.464,-0.197]	[-0.467,-0.195]
Having children			0.165*	0.215**
			[-0.001,0.331]	[0.046,0.385]
Employed			−0.098	−0.093
			[-0.228,0.033]	[-0.226,0.041]
Education				
*Less than high school*			(ref.)	(ref.)

*High school/some college*			−0.295***	−0.348***
			[-0.428,-0.162]	[-0.487,-0.210]
*College or higher*			−0.561***	−0.589***
			[-0.786,-0.336]	[-0.822,-0.357]
Homeowner			−0.024	−0.041
			[-0.157,0.108]	[-0.177,0.095]
Moved in the past five years			0.034	−0.002
			[-0.104,0.172]	[-0.143,0.139]
Own automobiles			−0.066	−0.097
			[-0.212,0.081]	[-0.248,0.053]
Own motorcycles			0.351	0.335
			[-0.152,0.854]	[-0.179,0.848]
***Neighborhood characteristics***				
Frequent violent crimes in neighborhood				0.467***
				[0.346,0.588]
Abandoned buildings within three blocks				0.120*
				[-0.014,0.254]
Landfills within three blocks				0.296***
				[0.154,0.438]
Poorly lit street within three blocks				0.044
				[-0.087,0.176]
Hospitals within 10 min’ walk				−0.142**
				[-0.279,-0.005]
Schools within 10 min’ walk				−0.124*
				[-0.258,0.011]
Sidewalks within block				0.097
				[-0.050,0.243]
Living in detached house				−0.001
				[-0.158,0.157]
Parks within 10 min’ walk				−0.072
				[-0.202,0.058]
Living in informal settlements				−0.939***
				[-1.140,-0.738]
No formal/informal transit stops within 10 min’ walk				0.339***
				[0.171,0.507]
Constant	−0.550***	−0.719***	−1.251***	−1.129***
	[-0.811,-0.290]	[-1.022,-0.416]	[-2.022,-0.480]	[-1.945,-0.313]
Variance of random intercept	0.170**	0.162**	0.190**	0.193**
	[0.018,0.322]	[0.016,0.308]	[0.021,0.359]	[0.018,0.368]
Intraclass correlation coefficient	0.049	0.047	0.055	0.055
	[0.021, 0.112]	[0.020, 0.108]	[0.023, 0.123]	[0.023, 0.127]


Number of observations	5,438	5,438	5,438	5,438
Number of groups	11	11	11	11
AIC	7002.3	7003.0	6901.3	6713.9

Note: Logistic regressions with random intercepts by city, ***p<0.01, **p<0.05, *p<0.1, 95% confidence interval in brackets.

**Table 4:** Associations between free-flow time/traffic delay and self-reported depressive symptoms (n = 5438).

Depressive symptoms	(5)	(6)	(7)	(8)
	Unadjusted	(5) + modes	(6) + socio-demographics	(7) + neighborhood characteristics
***Commuting patterns***				
Traffic delay in commute (10 min)	0.039**	0.050***	0.052***	0.040**
	[0.005,0.074]	[0.014,0.086]	[0.015,0.088]	[0.002,0.077]
Commuting time if no traffic (10 min)	−0.003	0.006	0.012	0.016
	[-0.030,0.025]	[-0.023,0.035]	[-0.017,0.041]	[-0.015,0.046]
Commuting modes				
*Private automobile*		(ref.)	(ref.)	(ref.)
				
*Formal transit*		0.072	−0.182	−0.239**
		[-0.125,0.269]	[-0.407,0.043]	[-0.468, −0.011]
*Informal transit*		0.237	−0.003	−0.071
		[-0.057,0.531]	[-0.320,0.314]	[-0.393,0.251]
*Taxi/Uber/Shuttle*		0.245	0.092	−0.002
		[-0.106,0.595]	[-0.271,0.456]	[-0.373,0.369]
*Non-motorized*		0.265**	−0.044	−0.106
		[0.052,0.478]	[-0.286,0.199]	[-0.353,0.140]
*Other*		0.232	0.037	−0.080
		[-0.061,0.525]	[-0.277,0.351]	[-0.400,0.241]
***Socio-demographic characteristics***				
Female			0.363***	0.356***
			[0.239,0.487]	[0.229,0.483]
Age			0.044**	0.032
			[0.007,0.082]	[-0.006,0.070]
Age squared			−0.0005**	−0.0003
			[-0.001,0.000]	[-0.001,0.000]
Living with spouse			−0.330***	−0.331***
			[-0.463,-0.197]	[-0.467,-0.195]
Having children			0.165*	0.215**
			[-0.001,0.331]	[0.046,0.384]
Employed			−0.099	−0.093
			[-0.230,0.032]	[-0.227,0.041]
Education				
*Less than high school*			(ref.)	(ref.)
				
*High school/some college*			−0.296***	−0.348***
			[-0.429,-0.163]	[-0.486,-0.210]
*College or higher*			−0.562***	−0.589***
			[-0.787,-0.337]	[-0.821,-0.357]
Homeowner			−0.026	−0.042
			[-0.159,0.106]	[-0.178,0.093]
Moved in the past five years			0.031	−0.004
			[-0.106,0.169]	[-0.145,0.137]
Own automobiles			−0.068	−0.098
			[-0.215,0.078]	[-0.249,0.052]
Own motorcycles			0.355	0.337
			[-0.148,0.859]	[-0.176,0.850]
***Neighborhood characteristics***				
Frequent violent crimes in neighborhood				0.465***
				[0.344,0.586]
Abandoned buildings within three blocks				0.118*
				[-0.017,0.252]
Landfills within three blocks				0.297***
				[0.155,0.438]
Poorly lit street within three blocks				0.045
				[-0.086,0.176]
Hospitals within 10 min’ walk				−0.142**
				[-0.279,-0.005]
Schools within 10 min’ walk				−0.124*
				[-0.259,0.011]
Sidewalks within block				0.097
				[-0.049,0.244]
Living in detached house				−0.001
				[-0.157,0.157]
Parks within 10 min’ walk				−0.073
				[-0.203,0.057]
Living in informal settlements				−0.934***
				[-1.135,-0.732]
No formal/informal transit stops within 10 min’ walk				0.340***
				[0.172,0.508]
Constant	−0.541***	−0.714***	−1.228***	−1.116***
	[-0.801,-0.280]	[-1.017,-0.412]	[-1.999,-0.456]	[-1.932,-0.299]
Variance of random intercept	0.169**	0.162**	0.190**	0.193**
	[0.018,0.321]	[0.016,0.308]	[0.021,0.358]	[0.018,0.367]
Intraclass correlation coefficient	0.049	0.047	0.054	0.055
	[0.021, 0.112]	[0.020, 0.108]	[0.023, 0.123]	[0.023, 0.127]


Number of observations	5,438	5,438	5,438	5,438
Number of groups	11	11	11	11
AIC	7001.8	7002.4	6901.2	6715.1

Note: Logistic regressions with random intercepts by city, ***p<0.01, **p<0.05, *p<0.1, 95% confidence interval in brackets.

In both sets of models, being a pedestrian and/or a cyclist appears to be associated with a higher probability of screening positively for depression (Models 2 and 6) than being a private automobile driver. Yet, this association completely disappears when we adjust for socio-demographics (Models 3 and 7). By contrast, in the fully adjusted models (Models 4 and 8), taking formal transit is associated with lower probability of depression than commuting by private automobiles. Specifically, the marginal effects suggest that formal transit users are 4.8% less likely to report depressive symptoms than private automobile commuters (Model 8).

### 4.3 Associations of socio-demographic and neighborhood characteristics

**Fig. 1:**
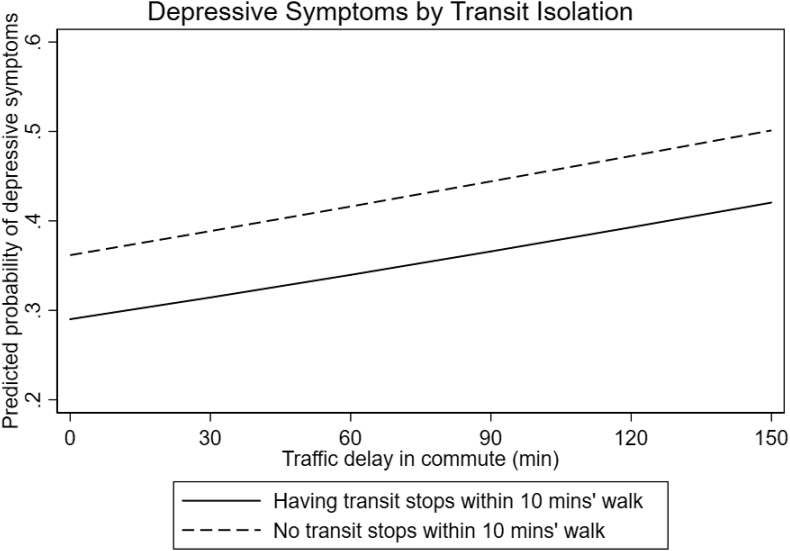
Predicted probability of depressive symptoms across traffic delay time, by transit isolation.

Both sets of models (Table 3, Table 4) also suggest that depression is significantly associated with particular socio-economic characteristics and neighborhood characteristics. Living in a neighborhood with violent crime, close to landfills, with abandoned buildings close by, and with the closest hospital more than 10 min away walking is associated with depression. Surprisingly, residents of informal settlements are less likely to be depressed, all else held equal. Finally, those with no formal/informal transit stops within a 10-min walk have a higher probability of depression (Fig. 1).

### 4.4 Validation of self-reported data

**Fig. 2:**
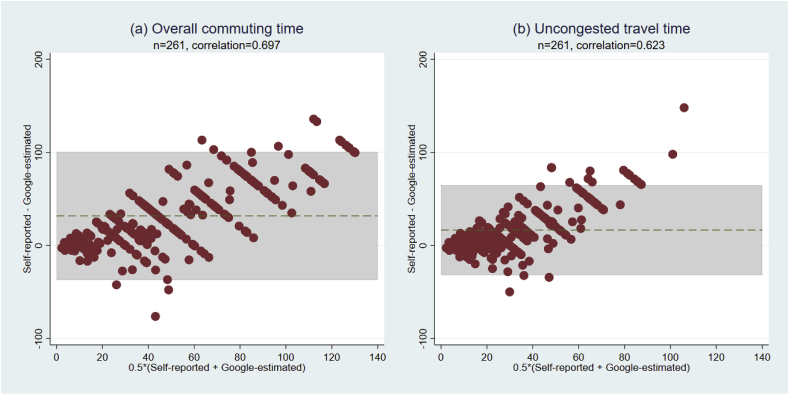
Bland-Altman plots for respondents in Bogotá (n = 261). Note: For overall commuting time, the Google-estimated time is the median value of 15 weekdays in May 2018; the uncongested travel time does not vary by day.

The validation of self-reported overall commuting time and uncongested travel time are summarized by Bland-Altman plots and Pearson’s correlations (Fig. 2). The Pearson’s correlation for overall commuting time and uncongested travel time are 0.697 and 0.623, respectively, suggesting moderate agreement. The Bland-Altman plots show that respondents systematically over-report actual and uncongested travel times, but the difference appears to change as commuting time changes. For lower values, participants under-reported their times, while this effect reversed for higher values. Three sources have the potential of reducing agreement between self-reported and Google-estimated travel times. First, the Google-estimated travel time is based on private automobiles, but only 71 of the 261 individuals reported using motorized modes (excluding BRT and rail transit with dedicated right-of-way) during their commutes. Second, the self-reported travel time are door-to-door time, while the Google-estimated travel time only considers in-vehicle travel time. Third, the Google-estimated travel time uses the centroid of 500m × 500m gridcells as opposed to the true origins and destinations, which might have made the estimation less accurate. Finally, variations in travel demand during different months of the year might have also contributed to the difference.

### 4.5 Sensitivity analyses

Several sensitivity analyses were conducted to test the robustness of our findings and several analytical decisions made. Instead of random effects models, we estimated fixed effects models with standard errors clustered by city for all eight models, and the coefficients and level of statistical significance for overall commute time, traffic delay time and commute modes did not differ. In addition, we estimated a random effects model with the raw CESD-10 score (ranging from 0 to 30) as a continuous dependent variable instead of the binary variable, and results were very similar to those presented here. We also tested two additional methods to identify the main commute modes, and the findings remain consistent.^2^ And we examined whether that commuting time may be non-linearly associated with depressive symptoms, but did not find significant non-linear associations.

We changed the thresholds for excluding observations with large delay-to-overall-time ratio to 85% and 95% (as opposed to 90% reported previously), and results remain mostly unchanged. We also changed the truncating values for overall commuting time and uncongested travel time to 150 and 210 min (as opposed to 180 min reported previously). For the 210-min-threshold models, the coefficients of overall commuting time and traffic delay time are significantly different from zero and did not differ from those in the 180-min-threshold models presented in Table 2 and Table 3. For the 150-min-threshold models, the coefficient of overall commuting time is significantly different from zero and similar to its counterpart in the 180-min-threshold model. However, the coefficient of traffic delay time in this set of model (0.031) is smaller than that in Table 3, and not significantly different from zero (p-value = 0.122).

To account for the heterogeneity of the 11 cities in population sizes, we re-ran the fully-adjusted models with two additional control variables: metropolitan-level population size and its square. Neither of these two terms are significantly different from zero. City-specific fully-adjusted models show that overall commuting time and traffic delay time were significant for models in cities with larger sample sizes (Bogotá, Caracas and Sao Paulo), while the effects are not significant for other cities (see Appendix).

## 5 Discussion

This study finds statistically significant associations between commuting patterns, travel options, and mental health in 11 Latin American cities. Commuters who take a longer time in their journey to their main activity (work, school, or other) are more likely to be screened positively for depression. In addition, this study takes one step further and disentangles the effect of overall commuting time. Commuters facing longer traffic delays – rather than longer uncongested travel time – during their journeys are more likely to have depression. These findings support and further expand the impedance theory by suggesting that the quality of commuting time is more important than the absolute value of travel time. This study also finds the benefit of use and access to mass transit for mental health. First, users of formal transit such as subways, BRT or buses are less likely to have depression than drivers. Second, those without good transit access are more likely to have depression.

The share of respondents being screened positively for depression in our study sample, 37%, is comparable with other research in the literature. Other studies in Latin America have estimated similar prevalence of positively screens for depressive symptoms (Batistoni et al., 2007; Wolf et al., 2002).

The findings of traffic delay connect to early work of the negative effects of highway congestion on mental health due to a lower sense of control (Schaeffer et al., 1988), lower predictability of the trip duration (Kluger, 1998) and a higher level of fatigue (Costa et al., 1988). All of these will create higher subjective impedance and make the commuters more likely to be in a depressive mood (Novaco et al., 1990).

This study also finds the benefit of using and accessing to mass transit for mental health. First, users of formal transit such as subways, BRT or buses are less likely to have depression than drivers. This finding is in concordance with the findings of Mytton et al. (2016). It is worth noting that drivers have the same propensity of depression as formal transit takers when not adjusting for socio-economic and neighborhood characteristics. It may because of the fact that drivers are more likely to have higher education level, to own a vehicle, and to live in a neighborhood with streetlights. All of them are correlated with better mental health. It may also be that the effects we identify for transit are moderated by the perceived quality of service. For example, individuals that perceive their city transit service to be poor or of low quality and who use transit may exhibit positive, not negative associations with the presence of depressive symptoms. We explored this by including a variable measuring perceived quality of transit service and interacting this variable with transit use but the coefficients for these new variables were not statistically significant (results not shown).

A second finding related to transit suggests that the study participants who reported not having mass transit options, formal or informal, within 10 minutes' walk from home were more likely to be screened positively for depressive symptoms. This is consistent with the hypothesis of Lucas (2012) that transport exclusion is associated with social exclusion, and hence potential mental health issues. It is noteworthy that the magnitude of the marginal effect of transit exclusion (4.8%) is considerably higher than the marginal effect of reducing travel delay time by 10 minutes (0.8%), even more so considering that the average delay time reported was 13 minutes. Thus, it appears that having transit within a 10-min walk of residence may provide more mental health dividends than simply reducing congestion. This is important for cities in a geographic area like Latin America which is consistently characterized by considerable traffic congestion, or that having transit within walking distance can proxy for other environmental attributes (such as sidewalks, improved lighting, and more activity).

Distance to transit, a proxy for local access to transit, is a crude descriptor of the benefits that may come from improved transit accessibility. The transit system ought to also connect the traveler with destinations, within a reasonable travel time, and at a reasonable cost. In our analyses, we controlled for actual mode used, in addition to the availability of transit close to home. Hence, the possible mental health benefits of local transit access identified are independent from the benefits that can emerge from using transit.

We did not find any significant associations between non-motorized modes and depression. This finding is different from Knott et al. (2018), but is similar to Chng et al. (2016) that transit connectivity matters more than active travel for mental health. That active transportation is not associated with depressive symptoms was unexpected given the existing evidence (LaJeunesse and Rodriguez, 2012; Mytton et al., 2016). One potential explanation is our way of defining the six mutually-exclusive travel modes: the “non-motorized” mode category refers to those only walking or bicycling. For example, a person using both bicycle and BRT to commute was categorized as “formal transit” in this study. To test this, we ran a model with an additional dummy variable identifying whether non-motorized travel modes were used for any segment of the trip. This dummy variable was not statistically significant at a 5% level of confidence, although the sign is negative (p = 0.072; results not shown). In lower- and middle-income country contexts, it may be that such associations are confounded by urban environmental factors such as quality of the infrastructure, air quality and noise. Another explanation is that, in many cities in Latin America, contrary to those of high income countries, active commuting is more need-based rather than by choice: a significant proportion of the population walk because they have no other mobility options for mobility (Salvo et al., 2014). For instance, in Bogotá, walking for transport has shown an inverse but not statistically significant pattern with health-related quality of life (Sarmiento et al., 2010).

In addition, this study found that low quality of social and built environment associates with higher probability of screening positively for depression. This agrees with several non-transportation studies focusing on the effects of neighborhood-level socio-economic and built-environment characteristics (e.g. Sampson et al. (2018) and Sarmiento et al. (2010)). Counterintuitively, commuters living in informal settlement are less likely to be depressed that those in formal settlements. One explanation is that communities in informal settlements might be more cohesive, making their residents less likely to exhibit depressive symptoms (Echeverría, 2008; Salcedo, 2010). Another explanation is that the communities in informal settlements might be less “structured and disciplined” hence exhibiting lower stress than communities in formal settlements (Izutsu et al., 2006). Admittedly, the literature of informal settlement and mental health is still limited, and more studies examining the mechanisms linking urban informality and mental health are needed.

This study has the following limitations, many of which should motivate further research. First, due to the cross-sectional nature of the dataset, we can only explain the relationship as associations rather than causation. It is possible that there is simultaneity between mental health and patterns of commute. Second, the overall commuting time, uncongested travel time and travel delay time are all self-reported. Our validations show an only moderate agreement between the two. Third, the dataset does not have variables on total income, either in household or individual level. Admittedly, collecting information on such variables is difficult in such a multinational survey and the irregularity of income per month of the studied population. We have included many of the variables which are able to reflect people’s income and socioeconomic status, such as education, employment, home ownership and neighborhood patterns. Finally, even though the surveyed individuals included and excluded in our study sample were comparable, they may be systematically different in unobserved ways that may be associated with the exposures and the outcome.

## 6 Conclusions

Using self-reported survey data from 11 Latin American cities, we found that longer commuting time is associated with higher probability of screening positively for depression. When examining differences by travel mode, users of formal transit are less likely to have depression than drivers. Furthermore, not having transit service within a 10-min walk from home is associated with depression. When the variable “commuting time” is unpacked into its components – uncongested travel time and congested delay time, we find that the delay induced by traffic congestion, rather than the time it takes to cover a distance uncongested, is associated with depression. However, these findings are likely to be driven by cities with larger sample sizes such as Bogotá, Caracas and Fortaleza.

The findings support the importance of mobility policies and individual mobility choices as determinants of mental health. Policy approaches that expand transit coverage, encourage transit use, and relieve congestion may also yield mental health benefits. Examples of these approaches include transit subsidy programs, integrated land use and transportation planning, and congestion management approaches such as cordon or congestion pricing. However, simply expanding roads and highways is unlikely to have mental health benefits, as these approaches alone have been shown not to reduce congestion.

## Declaration of conflicting of interests

The authors declared no potential conflicts of interest with respect to the research, authorship, and/or publication of this article.

## Financial disclosure

This study is supported by the *SALURBAL* project. The Salud Urbana en América Latina (*SALURBAL*)/Urban Health in Latin America project is funded by the Wellcome Trust [205177/Z/16/Z].

## Figures and Tables

**Table A1:** Depressive symptoms and commuting time: city-specific logit models

Depressive symptoms	BA	LAP	SP	FOR	BOG	QUI	LIM	MVD	CCS	PAC	MEX
***Commuting patterns***											
Commuting time (in 10 min)	−0.01	−0.02	0.05^∗^	0.01	0.05^∗∗^	0.04	0.05	0.07	0.08^∗∗^	−0.06	−0.05
	[-0.07,0.06]	[-0.11,0.06]	[-0.00,0.11]	[-0.17,0.19]	[0.01,0.10]	[-0.04,0.12]	[-0.02,0.12]	[-0.02,0.15]	[0.00,0.16]	[-0.18,0.05]	[-0.14,0.05]
Commuting modes											
*Private automobile*	(ref.)	(ref.)	(ref.)	(ref.)	(ref.)	(ref.)	(ref.)	(ref.)	(ref.)	(ref.)	(ref.)

*Formal transit*	−0.73^∗^	−0.17	−0.18	0.37	−0.18	−0.32	0.57	−0.39	−0.18	−0.59	−0.55
	[-1.47,0.01]	[-1.33,1.00]	[-0.70,0.34]	[-1.37,2.11]	[-0.80,0.45]	[-1.39,0.74]	[-0.57,1.72]	[-1.18,0.39]	[-0.99,0.63]	[-1.85,0.68]	[-1.52,0.42]
*Informal transit*		−0.34	−1.11		0.45	0.06	0.50	0.47	−0.46	1.67	0.76
		[-1.22,0.53]	[-3.21,0.98]		[-1.05,1.96]	[-1.89,2.01]	[-0.72,1.71]	[-0.81,1.76]	[-1.66,0.73]	[-0.53,3.87]	[-0.81,2.32]
*Taxi/Uber/Shuttle*		−0.93	0.42		0.63	1.80^∗^	0.73	−0.16		−0.84	0.12
		[-2.34,0.47]	[-0.60,1.44]		[-0.25,1.52]	[-0.14,3.74]	[-0.61,2.07]	[-1.96,1.64]		[-2.47,0.80]	[-1.19,1.43]
*Non-motorized*	−0.36	−0.61	−0.15	0.33	0.21	0.12	0.44	−0.31	−0.18	0.50	−0.33
	[-1.10,0.39]	[-1.60,0.38]	[-0.70,0.40]	[-1.54,2.21]	[-0.47,0.89]	[-1.22,1.45]	[-0.75,1.62]	[-1.14,0.52]	[-1.07,0.70]	[-1.28,2.28]	[-1.54,0.88]
*Other*	−0.99	0.95	−0.08	−0.72	−0.41	−0.22	0.86	0.21	0.15		2.02^∗∗^
	[-2.22,0.23]	[-1.17,3.06]	[-0.98,0.82]	[-2.79,1.35]	[-1.29,0.47]	[-2.43,1.99]	[-0.37,2.08]	[-0.84,1.27]	[-0.95,1.24]		[0.03,4.00]
***Sociodemographic characteristics***											
Female	0.46^∗∗^	0.37	0.39^∗∗^	0.06	0.36^∗∗^	0.35	0.42^∗^	0.53^∗∗^	0.12	0.68	0.08
	[0.06,0.86]	[-0.09,0.83]	[0.03,0.74]	[-0.87,0.99]	[0.01,0.71]	[-0.22,0.92]	[-0.01,0.84]	[0.09,0.98]	[-0.27,0.52]	[-0.19,1.55]	[-0.58,0.73]
Age	0.10	0.04	0.02	0.09	0.04	0.09	−0.06	0.03	0.08	−0.01	−0.08
	[-0.02,0.21]	[-0.10,0.19]	[-0.09,0.13]	[-0.21,0.40]	[-0.06,0.14]	[-0.09,0.27]	[-0.17,0.05]	[-0.10,0.15]	[-0.05,0.21]	[-0.26,0.23]	[-0.30,0.14]
Age squared	−0.001	−0.0003	−0.0003	−0.001	−0.001	−0.001	0.001	−0.0003	−0.001	0.0002	0.011
	[-0.00,0.00]	[-0.00,0.00]	[-0.00,0.00]	[-0.00,0.00]	[-0.00,0.00]	[-0.00,0.00]	[-0.00,0.00]	[-0.00,0.00]	[-0.00,0.00]	[-0.00,0.00]	[-0.00,0.00]
Living with spouse	−0.68^∗∗∗^	−0.96^∗∗∗^	−0.46^∗∗^	−0.68	−0.34^∗∗^	−0.21	−0.12	−0.33	−0.27	−0.08	0.47
	[-1.09,-0.26]	[-1.58,-0.34]	[-0.83,-0.08]	[-1.75,0.39]	[-0.68,-0.00]	[-0.87,0.45]	[-0.56,0.33]	[-0.77,0.11]	[-0.70,0.17]	[-0.93,0.77]	[-0.42,1.36]
Having children	0.36	0.97^∗∗∗^	0.23	−0.76	0.09	−0.11	0.47	0.51^∗^	−0.27	0.67	−0.64
	[-0.19,0.91]	[0.23,1.71]	[-0.21,0.67]	[-2.22,0.70]	[-0.35,0.53]	[-0.97,0.76]	[-0.10,1.05]	[-0.06,1.07]	[-0.80,0.25]	[-0.34,1.68]	[-1.65,0.38]
Employed	−0.58^∗∗∗^	−0.02	−0.29	−0.22	−0.17	−0.13	−0.11	−0.30	0.19	0.14	0.01
	[-1.00,-0.17]	[-0.52,0.48]	[-0.67,0.09]	[-1.50,1.06]	[-0.51,0.18]	[-0.71,0.45]	[-0.52,0.31]	[-0.74,0.14]	[-0.25,0.64]	[-0.82,1.10]	[-0.73,0.74]
Education											
*Less than high school*	(ref.)	(ref.)	(ref.)	(ref.)	(ref.)	(ref.)	(ref.)	(ref.)	(ref.)	(ref.)	(ref.)

*High school/some college*	−0.23	−0.73^∗∗^	−0.47^∗∗^	0.29	−0.62^∗∗∗^	−1.06^∗∗∗^	−0.40^∗^	−0.06	0.10	−0.38	0.03
	[-0.62,0.15]	[-1.36,-0.09]	[-0.86,-0.09]	[-0.80,1.37]	[-0.99,-0.24]	[-1.65,-0.47]	[-0.88,0.07]	[-0.53,0.40]	[-0.37,0.58]	[-1.38,0.62]	[-0.68,0.73]
*College or higher*	−0.45	−0.98^∗∗∗^	−0.49		−0.90^∗∗∗^	−1.23^∗∗^	−1.09^∗∗∗^	−1.01^∗^	−0.20	−0.42	−0.73
	[-1.73,0.84]	[-1.73,-0.24]	[-1.12,0.13]		[-1.52,-0.28]	[-2.43,-0.04]	[-1.90,-0.28]	[-2.04,0.02]	[-0.99,0.59]	[-1.66,0.83]	[-2.00,0.55]
Homeowner	0.03	−0.06	0.03	−0.12	0.04	0.03	−0.56^∗∗^	0.06	0.04	1.17^∗∗^	−0.02
	[-0.37,0.43]	[-0.55,0.43]	[-0.34,0.41]	[-1.25,1.01]	[-0.33,0.40]	[-0.57,0.62]	[-1.00,-0.12]	[-0.36,0.49]	[-0.52,0.60]	[0.11,2.22]	[-0.80,0.76]
Moved in the past five years	0.04	0.32	−0.23	0.19	−0.02	0.10	0.10	−0.16	−0.35	1.32^∗∗∗^	−0.69
	[-0.41,0.49]	[-0.17,0.80]	[-0.61,0.15]	[-1.02,1.39]	[-0.38,0.33]	[-0.47,0.67]	[-0.38,0.57]	[-0.59,0.27]	[-0.98,0.27]	[0.35,2.30]	[-1.61,0.22]
Own automobiles	−0.40	−0.40	−0.06	0.28	0.61^∗∗∗^	−0.05	−0.02	−0.57^∗∗^	−0.33	−0.62	0.03
	[-0.87,0.08]	[-0.88,0.08]	[-0.44,0.33]	[-1.30,1.85]	[0.16,1.06]	[-0.71,0.62]	[-0.54,0.49]	[-1.09,-0.05]	[-0.90,0.23]	[-1.65,0.40]	[-0.66,0.71]
Own motorcycles	−0.39	−0.52	0.31		1.14	1.33	1.64	0.14		2.11^∗^	0.60
	[-2.63,1.85]	[-2.02,0.99]	[-1.18,1.81]		[-0.35,2.62]	[-0.82,3.48]	[-0.81,4.09]	[-1.61,1.89]		[-0.30,4.53]	[-1.07,2.27]
***Neighborhood characteristics***											
Frequent violent crimes in neighborhood	0.55^∗∗^	0.70^∗∗∗^	0.67^∗∗∗^	1.43^∗∗∗^	0.31^∗^	0.48^∗^	0.44^∗∗^	0.85^∗∗∗^	0.16	0.88^∗^	−0.07
	[0.13,0.97]	[0.28,1.13]	[0.33,1.01]	[0.42,2.44]	[-0.00,0.63]	[-0.05,1.01]	[0.07,0.81]	[0.41,1.28]	[-0.24,0.56]	[-0.01,1.77]	[-0.67,0.54]
Abandoned buildings within three blocks	−0.04	0.19	0.07	−0.56	0.07	0.27	−0.14	0.13	0.09	1.50^∗∗∗^	0.20
	[-0.43,0.36]	[-0.27,0.65]	[-0.33,0.46]	[-1.81,0.69]	[-0.29,0.44]	[-0.31,0.85]	[-0.57,0.28]	[-0.28,0.54]	[-0.64,0.83]	[0.64,2.37]	[-0.49,0.89]
Landfills within three blocks	0.29	0.31	0.40	0.72	0.20	0.71^∗∗^	0.48^∗∗^	0.02	−0.14	−0.87	0.65
	[-0.14,0.71]	[-0.13,0.75]	[-0.13,0.93]	[-0.50,1.93]	[-0.17,0.58]	[0.11,1.31]	[0.02,0.93]	[-0.41,0.45]	[-0.62,0.35]	[-1.92,0.17]	[-0.20,1.49]
Poorly lit street within three blocks	0.47^∗^	0.54^∗∗^	0.22	−1.03^∗∗^	0.45^∗∗^	−0.31	−0.09	0.22	−0.27	0.87^∗∗^	−1.33^∗∗∗^
	[-0.00,0.94]	[0.08,1.00]	[-0.17,0.61]	[-2.06,-0.01]	[0.10,0.79]	[-0.88,0.26]	[-0.48,0.30]	[-0.20,0.65]	[-0.70,0.17]	[0.00,1.75]	[-1.97,-0.69]
Hospitals within 10 min’ walk	0.04	−0.38	−0.33	−0.03	−0.22	−0.51	−0.26	−0.32	0.01	0.56	0.17
	[-0.36,0.44]	[-0.90,0.14]	[-0.74,0.08]	[-1.01,0.94]	[-0.62,0.17]	[-1.13,0.11]	[-0.65,0.13]	[-0.80,0.16]	[-0.50,0.52]	[-0.40,1.52]	[-0.57,0.92]
Schools within 10 min’ walk	−0.30	−0.02	−0.11	1.01^∗^	−0.10	0.21	0.24	−0.52^∗∗^	−0.03	0.71	−0.71^∗∗^
	[-0.79,0.19]	[-0.50,0.47]	[-0.46,0.25]	[-0.15,2.16]	[-0.42,0.22]	[-0.38,0.79]	[-0.18,0.66]	[-1.01,-0.04]	[-0.56,0.51]	[-0.18,1.60]	[-1.38,-0.04]
Sidewalks within block	0.10	−0.10	−0.60	0.21	0.23	0.74^∗^	−0.08	0.06	0.26	−0.85^∗^	1.28^∗∗∗^
	[-0.36,0.56]	[-0.63,0.42]	[-1.82,0.63]	[-1.28,1.70]	[-0.11,0.57]	[-0.12,1.61]	[-0.60,0.43]	[-0.49,0.61]	[-0.16,0.68]	[-1.71,0.02]	[0.61,1.94]
Living in detached house	−0.58^∗∗^	0.72^∗∗^	0.06	0.00	0.28	−0.02	0.52^∗^	−0.03	−0.46^∗^	0.52	−1.18^∗∗∗^
	[-1.09,-0.07]	[0.06,1.38]	[-0.41,0.52]	[0.00,0.00]	[-0.11,0.66]	[-0.78,0.74]	[-0.04,1.07]	[-0.56,0.51]	[-0.94,0.03]	[-0.48,1.53]	[-1.94,-0.43]
Parks within 10 min’ walk	−0.49^∗∗^	0.65^∗∗∗^	0.17	−0.65	0.09	−0.44	−0.45^∗∗^	0.35	−0.78^∗∗∗^	−0.36	0.40
	[-0.89,-0.09]	[0.17,1.13]	[-0.19,0.54]	[-1.71,0.40]	[-0.24,0.42]	[-1.00,0.11]	[-0.86,-0.05]	[-0.17,0.87]	[-1.26,-0.30]	[-1.17,0.45]	[-0.28,1.08]
Living in informal settlements	−1.39^∗∗∗^	0.00	0.00	−0.03	−0.94^∗∗∗^				−0.38		
	[-1.89,-0.89]	[0.00,0.00]	[0.00,0.00]	[-1.01,0.94]	[-1.34,-0.54]				[-0.89,0.13]		
No transit stops within 10 min’ walk	0.10	0.23	0.52^∗^	2.27	0.41^∗∗^	0.11	0.54^∗^	−0.55	0.29	0.21	0.66
	[-0.54,0.75]	[-0.29,0.74]	[-0.01,1.04]	[-0.80,5.34]	[0.04,0.77]	[-0.54,0.75]	[-0.03,1.12]	[-1.62,0.52]	[-0.21,0.79]	[-1.06,1.47]	[-0.25,1.56]
Constant	−1.04	−1.73	0.15	−1.84	−1.64	−2.71	−0.44	−2.11	−1.10	−2.84	2.25
	[-3.47,1.38]	[-4.79,1.33]	[-2.29,2.59]	[-7.92,4.25]	[-3.72,0.45]	[-6.12,0.71]	[-2.97,2.08]	[-4.76,0.54]	[-3.61,1.42]	[-8.17,2.50]	[-1.77,6.28]

Number of observations	742	453	647	121	830	316	610	664	518	181	317
AIC	828.8	602.4	885.2	184.3	1059.4	426.0	791.8	680.3	702.6	238.0	369.1

Note: ***p<0.01, **p<0.05, *p<0.1, 95% confidence interval in brackets. Acronyms for cities: BA – Buenos Aires, LAP – La Paz, SP – Sao Paulo, FOR – Fortaleza, BOG – Bogotá, QUI – Quito, LIM – Lima, MVD – Montevideo, CCS – Caracas, PAC – Panamá City, MEX – México City.

**Table A2:** Depressive symptoms and congested/uncongested commuting time: city-specific logic models

Depressive symptoms	BA	LAP	SP	FOR	BOG	QUI	LIM	MVD	CCS	PAC	MEX
***Commuting patterns***											
Traffic delay (10 min)	0.003	0.06	0.10^∗^	−0.57^∗∗∗^	0.10^∗∗∗^	0.13	0.13	0.09	0.01	−0.00	−0.18^∗^
	[-0.13,0.14]	[-0.12,0.24]	[-0.02,0.21]	[-0.98,-0.15]	[0.02,0.17]	[-0.03,0.30]	[-0.03,0.29]	[-0.10,0.28]	[-0.15,0.17]	[-0.17,0.16]	[-0.37,0.01]
Uncongested time (10 min)	−0.02	−0.07	0.02	0.55^∗∗∗^	0.01	−0.04	−0.01	0.06	0.13^∗∗^	−0.19	0.05
	[-0.10,0.07]	[-0.20,0.05]	[-0.06,0.11]	[0.14,0.97]	[-0.05,0.08]	[-0.19,0.11]	[-0.13,0.11]	[-0.05,0.17]	[0.00,0.25]	[-0.46,0.09]	[-0.10,0.19]
Commuting modes											
*Private automobile*	(ref.)	(ref.)	(ref.)	(ref.)	(ref.)	(ref.)	(ref.)	(ref.)	(ref.)	(ref.)	(ref.)

*Formal transit*	−0.73^∗^	−0.16	−0.16	0.35	−0.18	−0.37	0.59	−0.41	−0.19	−0.56	−0.62
	[-1.47,0.02]	[-1.32,1.01]	[-0.69,0.36]	[-1.56,2.25]	[-0.80,0.45]	[-1.44,0.69]	[-0.56,1.74]	[-1.20,0.39]	[-1.01,0.62]	[-1.83,0.70]	[-1.60,0.35]
*Informal transit*		−0.35	−1.24		0.38	0.02	0.49	0.47	−0.47	1.56	0.82
		[-1.23,0.53]	[-3.41,0.93]		[-1.13,1.90]	[-1.94,1.97]	[-0.73,1.71]	[-0.82,1.75]	[-1.67,0.73]	[-0.65,3.77]	[-0.75,2.39]
*Taxi/Uber/Shuttle*		−0.96	0.41		0.64	1.79^∗^	0.71	−0.17		−0.80	0.03
		[-2.37,0.44]	[-0.62,1.43]		[-0.25,1.52]	[-0.17,3.75]	[-0.64,2.05]	[-1.97,1.63]		[-2.44,0.84]	[-1.29,1.35]
*Non-motorized*	−0.35	−0.58	−0.15	0.36	0.26	0.10	0.47	−0.31	−0.19	0.53	−0.41
	[-1.10,0.39]	[-1.58,0.41]	[-0.70,0.41]	[-1.65,2.37]	[-0.43,0.94]	[-1.24,1.44]	[-0.72,1.66]	[-1.14,0.52]	[-1.08,0.69]	[-1.26,2.31]	[-1.63,0.81]
*Other*	−0.99	1.05	−0.07	−0.52	−0.41	−0.20	0.87	0.21	0.13		1.97^∗^
	[-2.22,0.23]	[-1.06,3.17]	[-0.98,0.83]	[-2.76,1.72]	[-1.30,0.47]	[-2.43,2.03]	[-0.35,2.10]	[-0.84,1.26]	[-0.97,1.22]		[-0.04,3.99]
***Sociodemographic characteristics***											
Female	0.46^∗∗^	0.35	0.39^∗∗^	0.19	0.35^∗∗^	0.35	0.40^∗^	0.53^∗∗^	0.12	0.64	0.10
	[0.06,0.86]	[-0.11,0.81]	[0.03,0.74]	[-0.82,1.19]	[0.00,0.71]	[-0.22,0.92]	[-0.03,0.82]	[0.09,0.98]	[-0.28,0.52]	[-0.24,1.51]	[-0.56,0.76]
Age	0.10	0.05	0.02	0.16	0.04	0.09	−0.07	0.03	0.08	−0.01	−0.04
	[-0.02,0.21]	[-0.10,0.19]	[-0.09,0.13]	[-0.18,0.50]	[-0.06,0.14]	[-0.09,0.27]	[-0.18,0.05]	[-0.10,0.15]	[-0.04,0.21]	[-0.26,0.24]	[-0.27,0.18]
Age squared	−0.001	−0.0004	−0.0002	−0.002	−0.001	−0.001	0.001	−0.0003	−0.001	0.0001	0.001
	[-0.00,0.00]	[-0.00,0.00]	[-0.00,0.00]	[-0.00,0.00]	[-0.00,0.00]	[-0.00,0.00]	[-0.00,0.00]	[-0.00,0.00]	[-0.00,0.00]	[-0.00,0.00]	[-0.00,0.00]
Living with spouse	−0.68^∗∗∗^	−0.95^∗∗∗^	−0.45^∗∗^	−0.96	−0.34^∗∗^	−0.25	−0.13	−0.33	−0.26	−0.13	0.43
	[-1.09,-0.26]	[-1.57,-0.34]	[-0.82,-0.07]	[-2.15,0.23]	[-0.68,-0.00]	[-0.91,0.42]	[-0.57,0.32]	[-0.77,0.12]	[-0.69,0.18]	[-0.99,0.73]	[-0.47,1.33]
Having children	0.36	0.98^∗∗∗^	0.22	−0.86	0.08	−0.12	0.48	0.51^∗^	−0.26	0.68	−0.64
	[-0.19,0.91]	[0.24,1.71]	[-0.22,0.67]	[-2.43,0.71]	[-0.36,0.52]	[-0.99,0.75]	[-0.09,1.06]	[-0.06,1.08]	[-0.79,0.26]	[-0.33,1.70]	[-1.67,0.38]
Employed	−0.58^∗∗∗^	−0.02	−0.29	−0.36	−0.16	−0.12	−0.13	−0.30	0.20	0.06	0.03
	[-1.00,-0.16]	[-0.53,0.48]	[-0.67,0.09]	[-1.73,1.02]	[-0.50,0.19]	[-0.70,0.46]	[-0.55,0.29]	[-0.74,0.14]	[-0.25,0.65]	[-0.92,1.04]	[-0.71,0.77]
Education											
*Less than high school*	(ref.)	(ref.)	(ref.)	(ref.)	(ref.)	(ref.)	(ref.)	(ref.)	(ref.)	(ref.)	(ref.)

*High school/some college*	−0.24	−0.74^∗∗^	−0.48^∗∗^	0.28	−0.61^∗∗∗^	−1.05^∗∗∗^	−0.41^∗^	−0.06	0.11	−0.35	0.02
	[-0.62,0.15]	[-1.37,-0.10]	[-0.86,-0.09]	[-0.84,1.40]	[-0.99,-0.23]	[-1.64,-0.45]	[-0.88,0.07]	[-0.53,0.40]	[-0.36,0.59]	[-1.36,0.65]	[-0.69,0.73]
*College or higher*	−0.46	−0.99^∗∗∗^	−0.48		−0.89^∗∗∗^	−1.27^∗∗^	−1.11^∗∗∗^	−1.01^∗^	−0.20	−0.37	−0.82
	[-1.76,0.83]	[-1.74,-0.24]	[-1.11,0.14]		[-1.51,-0.26]	[-2.47,-0.06]	[-1.93,-0.30]	[-2.04,0.02]	[-0.99,0.59]	[-1.63,0.89]	[-2.10,0.45]
Homeowner	0.03	−0.07	0.04	−0.29	0.03	0.03	−0.56^∗∗^	0.06	0.04	1.22^∗∗^	0.13
	[-0.37,0.43]	[-0.56,0.42]	[-0.33,0.41]	[-1.52,0.94]	[-0.34,0.39]	[-0.57,0.62]	[-1.00,-0.12]	[-0.36,0.49]	[-0.52,0.60]	[0.15,2.29]	[-0.67,0.94]
Moved in the past five years	0.04	0.32	−0.25	0.30	−0.04	0.11	0.10	−0.16	−0.36	1.29^∗∗∗^	−0.59
	[-0.40,0.49]	[-0.16,0.80]	[-0.62,0.13]	[-0.94,1.54]	[-0.39,0.32]	[-0.46,0.69]	[-0.38,0.57]	[-0.60,0.27]	[-0.98,0.27]	[0.31,2.27]	[-1.52,0.34]
Own automobiles	−0.40	−0.39	−0.06	0.63	0.59^∗∗^	−0.09	−0.02	−0.57^∗∗^	−0.31	−0.60	−0.00
	[-0.87,0.08]	[-0.87,0.10]	[-0.45,0.32]	[-1.14,2.40]	[0.13,1.04]	[-0.75,0.58]	[-0.53,0.50]	[-1.09,-0.05]	[-0.88,0.26]	[-1.64,0.43]	[-0.69,0.69]
Own motorcycles	−0.40	−0.54	0.32		1.18	1.33	1.56	0.16		2.34^∗^	0.69
	[-2.64,1.85]	[-2.05,0.97]	[-1.16,1.81]		[-0.31,2.67]	[-0.86,3.52]	[-0.91,4.03]	[-1.59,1.91]		[-0.06,4.74]	[-0.99,2.36]
***Neighborhood characteristics***											
Frequent violent crimes in neighborhood	0.55^∗∗^	0.70^∗∗∗^	0.67^∗∗∗^	1.47^∗∗∗^	0.31^∗^	0.44	0.45^∗∗^	0.85^∗∗∗^	0.18	0.90^∗∗^	−0.06
	[0.13,0.97]	[0.28,1.13]	[0.33,1.01]	[0.39,2.55]	[-0.01,0.63]	[-0.10,0.97]	[0.08,0.82]	[0.41,1.28]	[-0.22,0.58]	[0.01,1.79]	[-0.67,0.55]
Abandoned buildings within three blocks	−0.04	0.17	0.05	−0.72	0.08	0.30	−0.16	0.13	0.13	1.51^∗∗∗^	0.24
	[-0.44,0.36]	[-0.29,0.63]	[-0.34,0.45]	[-2.04,0.60]	[-0.29,0.45]	[-0.28,0.88]	[-0.58,0.27]	[-0.28,0.54]	[-0.61,0.87]	[0.64,2.37]	[-0.45,0.94]
Landfills within three blocks	0.28	0.32	0.40	0.64	0.21	0.65^∗∗^	0.47^∗∗^	0.03	−0.12	−0.92^∗^	0.71
	[-0.15,0.71]	[-0.12,0.76]	[-0.13,0.93]	[-0.64,1.93]	[-0.17,0.59]	[0.05,1.26]	[0.01,0.93]	[-0.41,0.46]	[-0.61,0.36]	[-1.98,0.14]	[-0.14,1.56]
Poorly lit street within three blocks	0.47^∗∗^	0.54^∗∗^	0.21	−0.84	0.46^∗∗∗^	−0.31	−0.10	0.22	−0.26	0.89^∗∗^	−1.43^∗∗∗^
	[0.00,0.95]	[0.07,1.00]	[-0.18,0.61]	[-1.92,0.25]	[0.12,0.80]	[-0.89,0.26]	[-0.49,0.29]	[-0.21,0.65]	[-0.70,0.18]	[0.02,1.77]	[-2.09,-0.78]
Hospitals within 10 min’ walk	0.04	−0.38	−0.32	0.24	−0.23	−0.53^∗^	−0.26	−0.32	0.02	0.53	0.20
	[-0.36,0.44]	[-0.90,0.14]	[-0.73,0.09]	[-0.81,1.28]	[-0.63,0.16]	[-1.16,0.09]	[-0.65,0.14]	[-0.80,0.15]	[-0.48,0.53]	[-0.44,1.49]	[-0.56,0.95]
Schools within 10 min’ walk	−0.31	−0.03	−0.12	0.84	−0.09	0.23	0.24	−0.53^∗∗^	−0.04	0.75	−0.75^∗∗^
	[-0.80,0.19]	[-0.51,0.45]	[-0.47,0.24]	[-0.39,2.08]	[-0.41,0.23]	[-0.36,0.82]	[-0.18,0.65]	[-1.01,-0.04]	[-0.58,0.50]	[-0.15,1.64]	[-1.42,-0.08]
Sidewalks within block	0.10	−0.12	−0.60	−0.34	0.24	0.71	−0.10	0.07	0.24	−0.83^∗^	1.29^∗∗∗^
	[-0.36,0.56]	[-0.65,0.41]	[-1.82,0.63]	[-1.97,1.29]	[-0.10,0.58]	[-0.16,1.58]	[-0.62,0.42]	[-0.48,0.62]	[-0.18,0.66]	[-1.70,0.04]	[0.62,1.96]
Living in detached house	−0.58^∗∗^	0.73^∗∗^	0.06		0.28	−0.06	0.52^∗^	−0.03	−0.45^∗^	0.47	−1.24^∗∗∗^
	[-1.09,-0.07]	[0.06,1.39]	[-0.41,0.53]		[-0.10,0.67]	[-0.82,0.70]	[-0.03,1.07]	[-0.56,0.51]	[-0.93,0.04]	[-0.53,1.47]	[-2.00,-0.47]
Parks within 10 min’ walk	−0.49^∗∗^	0.68^∗∗∗^	0.18	−0.58	0.09	−0.47^∗^	−0.46^∗∗^	0.36	−0.78^∗∗∗^	−0.39	0.42
	[-0.89,-0.09]	[0.19,1.16]	[-0.18,0.54]	[-1.69,0.54]	[-0.24,0.42]	[-1.03,0.08]	[-0.87,-0.05]	[-0.16,0.88]	[-1.26,-0.30]	[-1.21,0.42]	[-0.26,1.10]
Living in informal settlements	−1.39^∗∗∗^			−0.45	−0.92^∗∗∗^				−0.41		
	[-1.89,-0.89]			[-1.50,0.61]	[-1.32,-0.52]				[-0.92,0.11]		
No transit stops within 10 min’ walk	0.11	0.24	0.53^∗∗^	2.75^∗^	0.41^∗∗^	0.08	0.54^∗^	−0.54	0.28	0.22	0.60
	[-0.54,0.76]	[-0.27,0.76]	[0.01,1.06]	[-0.26,5.77]	[0.04,0.78]	[-0.56,0.73]	[-0.04,1.11]	[-1.61,0.53]	[-0.22,0.79]	[-1.04,1.48]	[-0.32,1.51]
Constant	−1.04	−1.80	0.18	−2.92	−1.57	−2.41	−0.30	−2.12	−1.22	−2.88	1.53
	[-3.46,1.39]	[-4.86,1.27]	[-2.27,2.62]	[-9.69,3.85]	[-3.66,0.52]	[-5.86,1.05]	[-2.83,2.24]	[-4.77,0.53]	[-3.76,1.31]	[-8.25,2.48]	[-2.56,5.63]

Number of observations	742	453	647	121	830	316	610	664	518	181	317
AIC	830.8	603.3	886.4	173.1	1059.0	426.4	792.5	682.2	703.7	239.0	368.3

Note: ***p<0.01, **p<0.05, *p<0.1, 95% confidence interval in brackets. Acronyms for cities: BA – Buenos Aires, LAP – La Paz, SP – Sao Paulo, FOR – Fortaleza, BOG – Bogotá, QUI – Quito, LIM – Lima, MVD – Montevideo, CCS – Caracas, PAC – Panamá City, MEX – México City.

## Footnotes

1 The 14 mode options to select from included: (1) private automobile; (2) firm or educational establishment transportation; (3) minibus/jeeps/combis; (4) taxi; (5) Uber/app-based ride hailing; (6) motorcycle or bicycle taxi; (7) walking; (8) bicycle; (9) motorcycle; (10) subway; (11) suburban train; (12) buses/coaches (formal); (13) BRT; (14) others. To create the categorical variable, we used the following rule: (a) if 10, 11, 12 or 13: “formal transit”, otherwise, (b) if 3: “informal transit”, otherwise, (c) if 2, 4, or 5: “taxi/Uber/shuttle”, otherwise, (d) if 1: “private automobile”, otherwise, (e) if 7 or 8: “non-motorized”, otherwise: “other”.

2 The two alternative methods are: (a). Private automobiles, formal transit, informal transit, for-hire service (taxi/Uber), non-motorized, other; (b). Private automobiles, formal transit, informal transit, for-hire service (taxi/Uber), motorcycle, non-motorized, other. Compared to the original method demonstrated in Section 3.3, method (a) removed the "school/company shuttle" to “other”; compared to method (a), method (b) moved “motorcycles” from “other” to a stand-alone mode.
